# Transpulmonary Hypothermia with Cooled Oxygen Inhalation Shows Promising Results as a Novel Hypothermia Technique

**DOI:** 10.4274/balkanmedj.2016.0782

**Published:** 2017-05-15

**Authors:** Yahya Ayhan Acar, Banu Karakuş Yılmaz, Duygu Sultan Çelik, Erdem Çevik, Hatice Topçu, Şule Özsoy, Aylin Haklıgör, Orhan Çınar

**Affiliations:** 1 Clinic of Emergency Medicine, Etimesgut Military Hospital, Ankara, Turkey; 2 Clinic of Emergency Medicine, Hamidiye Şişli Etfal Training and Research Hospital, İstanbul, Turkey; 3 Research Center, Bağcılar Training and Research Hospital, İstanbul, Turkey; 4 Clinic of Emergency Medicine, Van Military Hospital, Van, Turkey; 5 Clinic of Pathology, Bağcılar Training and Research Hospital, İstanbul, Turkey; 6 Clinic of Biochemistry, Adana Numune Training and Research Hospital, Adana, Turkey; 7 Department of Emergency Medicine, Acıbadem University School of Medicine, İstanbul, Turkey

**Keywords:** Induced hypothermia, pulmonary ventilation, oxygen

## Abstract

**Background:**

Therapeutic hypothermia was showed to improve neurologic outcome but current therapeutic hypothermia techniques have limitations. Novel techniques such as transpulmonary hypothermia with cooled oxygen inhalation may be beneficial.

**Aims:**

To evaluate the performance of transthoracic hypothermia with cooled medical oxygen inhalation as a therapeutic hypothermia method.

**Study Design:**

Animal experimentation.

**Methods:**

A total of 36 adult male Wistar-Hannover rats were used in this research. Rats were randomised into four groups: group 1, Cooled oxygen group; group 2, IV cold fluid group; group 3, Surface cooling group; group 4, control group. No hypothermia method was applied in the control group. Hypothermia techniques were administered in the other three groups until the targeted core temperature was maintained. The target temperature was continued for one hour at 32-34 °C. After that, rats were heated up with hot blankets. Once the rectal temperature reached 38 °C, rats were euthanised. The main outcomes were the rate of temperature decrease (°C per minute) (S) and the time required to reach the target body temperature (T).

**Results:**

All rats survived the study protocol. When compared to the control group, T and S values were better in the cooled medical oxygen inhalation group (p<0.001). The IV cold fluid group had lower S values and higher T values compared to the cooled oxygen group (p<0.001, and p=0.003, respectively). There was no meaningful pathology in the histological samples in any group.

**Conclusion:**

As an easy-to-use and inexpensive method, cooled oxygen inhalation may be a beneficial hypothermia technique.

Therapeutic hypothermia (TH) is the term that describes interventions which decrease a patient’s core temperature to prevent secondary organ damage of a primary injury (1). TH has also been reported as a unique intervention that improves neurologic recovery in post-cardiac arrest care (2,3). The 2015 American Heart Association guidelines are recommending that TH is maintained between 32 and 36 °C stronger than the 2010 guidelines (3,4). Current guidelines also state that there is no contraindication to controlling the temperature between 32 and 36 °C (4). However, unfortunately, the gold standard technique for TH has not been defined and there are still debates about specific indications, patient groups, timing, hypothermia technique, and maintenance. As a known method, intravenous saline administration at +4 °C and surface cooling have been widely accepted as hypothermia techniques in clinical areas, but both have limitations. Moreover, current guidelines no longer recommend pre-hospital induced hypothermia with IV cold fluid administration because of possible harmful effects such as pulmonary oedema due to the large volume of fluid infusion (4,5). These data show that reversible and easy applicable TH techniques can be beneficial.

Transpulmonary hypothermia with cooled medical oxygen inhalation has potential advantages, such as being easily applicable, acting on the large capillary surface of the lungs that surround the heart muscle, and being reversible because of the continuous gas exchange in the capillary membranes. It can also be easily combined with other TH techniques. Recently, Kumar et al. (6) described a transpulmonary hypothermia model that uses a combination of gases in a porcine model for rapid brain cooling. Rather than a gas combination, the inhalation of cooled medical oxygen was hypothesised as a novel hypothermia technique in our previous reports (7,8).

In the current study, our research question is whether transthoracic hypothermia could be used as a TH technique with cooled oxygen inhalation. We used a rat model to compare the efficacy of transthoracic hypothermia with medical oxygen at +4 °C to current hypothermia techniques.

## MATERIAL AND METHODS

This is a randomised, controlled, examiner-blinded experimental study conducted with 36 adult Wistar-Hannover male rats weighing 300-500 grams. The main outcomes of this experimental study were the “rate of temperature decrease (°C/min)” of rectal temperature decrease (S) and the time required to reach the targeted body temperature (T) with the cooled oxygen inhalation technique. Primary experimental outcome was to assess the pathological changes in the lung, liver, and kidney tissues. The study protocol was approved by the local animal studies ethical committee. Rats were acquired from a nationally certified laboratory animal centre (certification number: 51; April 2^nd^, 2012). All rats were housed in the laboratory with a standard temperature of 22 °C, and in standard dark/light cycles. Animals were fed with 21% protein ingredient feed and had fresh water daily. All animals subjected to same instrumentation procedures as intubation, tail vein cannulation, and monitoring. Power analysis was calculated as 7 rats for each group for 80% of power and 0.05 of α error value.

**Figure 1:**
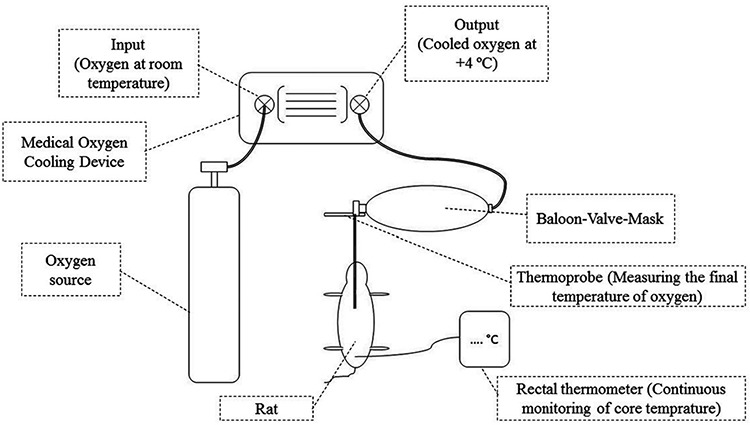
Schematic drawing of interventions and devices used during the study protocol.

Rats were anaesthetised with 50 mg/kg ketamine (Ketalar^®^; Pfizer, NY, USA) and 10 mg/kg xylazine (Rompun^®^; Bayer, ON, Canada) and were monitored (Datex-Ohmeda^®^; GE, Helsinki, Finland). Then, rats were randomised into four groups through the random number table created by a computer. Study groups were as follows:

• Group 1 (n=9): Cooled oxygen group. This group received cooled medical oxygen at +4 °C. Standard oxygen temperature was maintained with a cooling device designed for this purpose by researchers. A cooling device was designed to maintain medical oxygen in a standard temperature between +10 and -10 ºC. It used a 220 volt electric supply and medical oxygen. Medical oxygen received from the input was insufflated through a coiled tube system (serpentine) covered by a cooling fluid. The cooling device used an absorption refrigerator system and desired temperature of the oxygen was adjusted by digital control panel. During this procedure, medical oxygen was moved separately in the serpentine and did not make contact with any chemical or other material. Additionally, the cooling device was approved by standard certifications, calibrated by certified technicians, and assessed by board directors of the laboratory in which the study was conducted. Rats were intubated with a 16 G catheter and oxygen was given through a balloon-valve-mask (BVM) at a rate of 200 mL/min; the fractioned oxygen concentration was 1.0 (100% oxygen concentration). As an additional standardisation technique, a Thermoprobe TP7 (ThermoProbe Inc., Jackson, MS, United States of America) was placed at the tip of the BVM and final temperature of the oxygen was measured during whole procedures. Schematic drawing of the study protocol is given in Figure 1.

• Group 2 (n=9): Four cold fluid group. In this group, lactated ringer (LR) solution was given at +4 °C through the tail vein, as with the hypothermia technique. During the first hour, LR was administered at a rate of 30 mL/kg/h, and then at 10 mL/kg/h until the target temperature was maintained.

• Group 3 (n=9): Surface cooling group. Standardised evaporation technique was applied to this group with a cooling fan and ice packs stored at 0 °C.

• Group 4 (n=9): Control group. As the control group, no hypothermia procedure was applied to this group; the routine anaesthesia protocol was performed.

**Figure 2:**
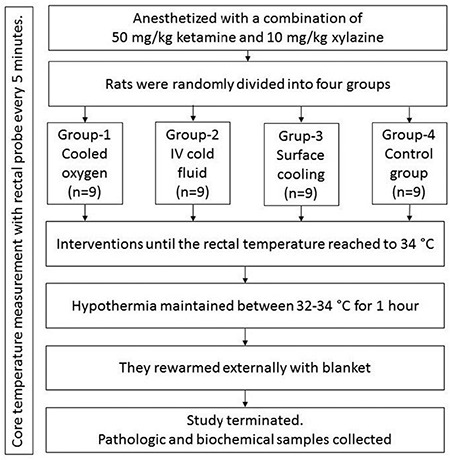
The time-line diagram of the study.

Rats were monitored for rectal temperature, pulse rate, and respiratory rate and temperature values were recorded every 5 minutes. Instrumentation was applied through the tail vein, interventions for TH were administered in groups 1 to 3 until core temperatures reached 34 °C, and the time required was recorded. TH interventions were administered for at least 60 minutes and if any rat did not reach the target temperature, the T value was recorded as 60 minutes. The target temperature was continued between 32 and 34 °C for one hour via defined TH techniques. Then, TH interventions were stopped and the rats were heated with blankets. When the rectal temperature of the rats reached 38 °C, total time was recorded and the rats were euthanised. Histological samples from the kidney, heart, and lung tissues were taken and stored in formaldehyde solution. Tissue and blood samples were blinded before examination. The timeline of the study is given in Figure 2.

### Biochemical analyses

Biochemical parameters including glucose, creatinine, alanine amino-transferase (ALT), alkaline phosphatase, blood urea nitrogen, potassium, sodium, and calcium were studied to assess the metabolic effects of different hypothermia techniques. Serum was obtained by centrifuging (5000 rpm for 5 minutes) blood samples collected from cardiac puncture and stored in a freezer (-20 °C) before analysis. Biochemical analyses were performed with a VetScan 2 test kit (Abaxis, Union City, CA, United States of America). All samples, including those for pathologic examination, were coded for blinding of the examiners.

### Pathological examinations

Rats were euthanised 2 hours after termination of the study protocol (reaching the rectal temperature of 38 °C) because neutrophil recruitment into the inflammation site occurs rapidly within the first 2 hours (9). Granular exocytosis is the mechanism of cell-derived mediator secretion, so mast cell stabilisation was evaluated to determine the degranulation ratio that might initiate inflammation (10).

Lung, liver, and kidney samples were fixed in 40 g/L paraformaldehyde, then frozen and cut into 5 µm slices for haematoxylin-eosin and toluidine blue staining. Light microscope examination was used to assess the hypoxia/ischaemia gradation. Mast cell degranulation ratio (degranulated/granulated) in the perivascular region was assessed as the first step of the inflammatory process.

### Statistical analysis

Descriptive statistics for categorical variables were presented as frequency and percentage. Kolmogorov-Smirnov test was used to test the normally distribution of the data. Data were presented as median ± interquartile range and minimum-maximum for non-normal data. The Kruskal-Wallis test was used for comparison of groups for T, S, and biochemical results. Values were analysed post hoc using the Mann-Whitney U test followed by Bonferonni correction. Statistical analyses were performed with Statistical Package for the Social Sciences version 15.0 (SPSS Inc., Chicago, IL, United States of America).

## RESULTS

**Figure 3:**
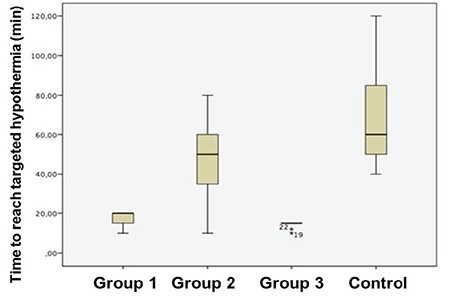
Cooled oxygen inhalation groups showed temperature decreases at higher rates compared to the control group.

**Figure 4:**
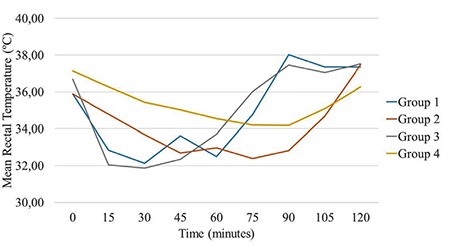
Mean rectal temperature changes of all groups in time (minutes). O_2_: transpulmonary hypothermia group; IV: intravenous cold fluid group; Ext: surface cooling group; Cont: control group

All of the rats survived the study protocol and no adverse effects were observed. Despite the randomisation prior to the study, groups showed a difference at the baseline temperatures (p=0.036). The control group never reached the target temperature of 34 °C. T (median: 20 minutes) and S (median: 0.20 °C/min) values were preferable in group 1, which had medical oxygen at 4 °C, compared to the control group (median for T: 60 minutes; median for S: 0.06 °C/min) (p<0.001). The cold LR group (group 2) had higher T (median: 50 minutes) values and lower S (median: 0.07 °C/min) values than the medical oxygen group (p=0.003 and p<0.001, respectively). S (median: 0.30 °C/min) values were higher in the surface cooling group than in the cooled medical oxygen group (median: 0.20 °C/min) and surface cooling group reached the targeted temperature faster than the cooled oxygen group (p=0.001). However, T (median: 15 minutes) values did not show a statistically significant difference between surface cooling and cooled oxygen (median: 20 minutes) groups (p=0.06). When compared to the control group (median for T: 60 minutes; median for S: 0.06 °C/min), T (median value: 50 minutes) and S (median value: 0.07 °C/min) values were no different in intravenous fluid group (p=0.94 and p=0.730, respectively). The surface cooling group showed a better performance than the IV cold fluid group in both T (median: 15 vs. 50 minutes and S (median: 0.07 vs. 0.30 °C/min) values (p=0.003 and p<0.001, respectively) (Table 1, Figure 3, Figure 4).

Group 2 (137.3±63.7) and 3 showed higher ALT levels compared to group 1 (150.7±61.19) and control (95.2±24.8) groups (p=0.011). Also, group 3 (132.6±3.67) showed lower sodium levels compared to other groups (p=0.008). There was no difference in other biochemical parameters between the four groups (Table 2).

**Figure 5:**
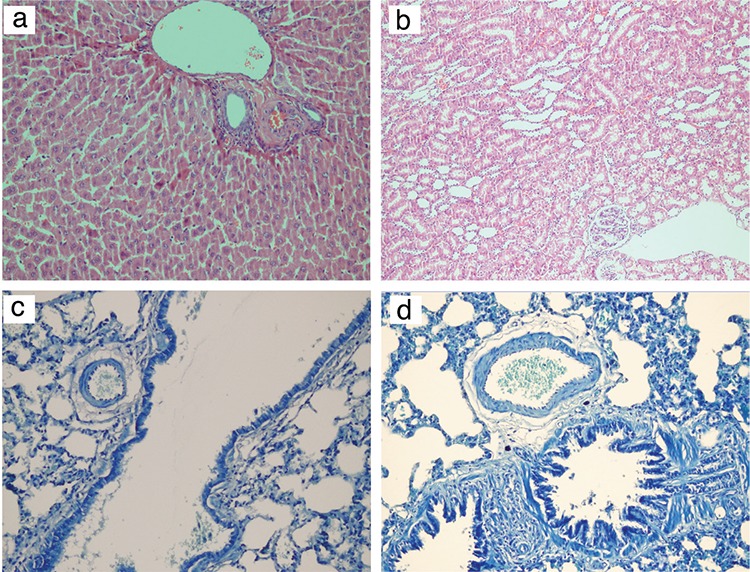
Histological examination of the kidney, liver, and lung tissues obtained from cooled oxygen group. Histopathological view of liver without any change (haematoxylin-eosin x100) (a). Renal tissue with normal glomeruli and tubules (haematoxylin-eosin x100) (b). Bronchial structures with slight inflammation (toluidine blue x100) (c). Minimal perivascular oedema in the lung tissue (toluidine blue x200) (d).

No pathological changes in histologic examination of the kidney and liver were observed in any of the groups. However, histopathological examination of the lung showed minimal perivascular oedema adjacent to the main bronchi, but no tissue damage in the cooled oxygen group (Figure 5).

## DISCUSSION

According to our study of a rat model, compared to current TH interventions, the inhalation of cooled medical oxygen seems efficient in reaching the target core temperature without any tissue damage.

Kumar et al. (6) recently introduced transthoracic hypothermia in a pig model using a perfluorocarbon aerosol and cooled helium-oxygen mixture. Our method uses mainly the same mechanism, but we used medical oxygen rather than a gas combination. Because transpulmonary hypothermia is a novel method, there is no consensus on which gas to use and/or at what temperature. Kumar et al. (6) used a gas combination at 0±2 °C. In this study, we tested cooled oxygen at +4 °C for intubated rats and ventilation with medical oxygen at +4 °C seemed effective as a TH technique.

Our data show that transpulmonary hypothermia with cooled medical oxygen inhalation is efficient for the rapid cooling phase-even more effective than IV fluid administration. If it could be transposed into clinical practice, this novel technique’s most important possible future effect could be to make TH interventions possible because it is rapid, non-invasive, and moreover, easily reversible because of the continuous gas exchange in the lungs (11,12).

Early initialisation of hypothermia using a combination of current techniques in the out-of-hospital environment has been suggested because of the possible benefits (13,14). However, current guidelines recommend against pre-hospital induced hypothermia with IV cold fluid administration due to possible side effects such as pulmonary oedema. Using cooled medical oxygen also has the potential to make initialising hypothermia in out-of-hospital settings possible. It can also be combined with current hypothermia techniques for better applications.

One of the pre-hospital hypothermia studies examined intranasal cooling versus no hypothermia and reported successful results (15). They used air or oxygen with a specific device. Although intra-nasal cooling has a much smaller surface area, it was shown to be effective in clinical use (15). Our technique is based on cooling with cold air though the lungs and the total surface of capillary alveolar area is enormous compared to nasal mucosa. Additionally, through alveolar capillary membranes, cooled oxygen can be transmitted to erythrocytes and act as a systemic cooling agent.

However, previous guidelines suggested to avoid prolonged hyperoxia during resuscitation, current guidelines suggest using oxygen with high concentrations for oxygenation of the patients after the return of spontaneous circulation to prevent hypoxia (4). Our technique allows oxygen to be given in higher concentrations because, according to Charles’ law, a decrease in the temperature will lead to an increase in the concentration of the oxygen molecules per unit area.

Biochemical analyses were performed to assess whether any technique would cause significant injury to the liver and kidney or cause any metabolic changes. Blood glucose levels were higher in all groups and did not show a difference between groups. Previous data suggest that the ketamine-xylazine combination may cause an acute hyperglycaemic response and that this effect will reach a maximum at 120 minutes (16). The ketamine-xylazine combination is also associated with mild ALT and AST elevations without any histopathological changes in the liver in rats (17). According to our results, ALT and sodium levels showed difference between groups. However these differences were not of clinical importance, with the cooled oxygen group showing a better result for both parameters.

Potential acute pathologic changes in the inflammatory cascade are expected to occur within one hour and, during the study protocol, TH was continued for one hour (9). In their study on transpulmonary hypothermia, Kumar et al. (6) reported that there was no evidence of pathological changes in the study groups. Larsson et al. (18) reported that cold air inhalation could increase inflammatory changes in the lung. However, in their study, healthy subjects inhaled cold air at -23 °C for 2 hours. Our study group received cold oxygen at 4 °C for 1 hour but no pathological changes occurred. These findings can show that the degree and duration of the exposure can affect alveolar injury. Additionally, alveolar injury including alveolar haemorrhage, alveolar neutrophil infiltration, alveolar macrophage proliferation, and interstitial congestion caused by high peak airway pressure and large tidal volume was reported (19). In our study, airway pressure was not applied and future studies on the effect of cooled oxygen in high pressure and high tidal volumes may be beneficial.

This experimental study has several limitations, including the absence of brain temperature measurement basal biochemical measurements. Despite randomisation, baseline temperatures showed differences between groups. That was concluded as a result of an extreme temperature (38.3 ºC) in one group, but was not associated with a direct effect of main outcomes (T and S values) which are based on continuous measurements by time. Additionally, as a primary study, rats were used to test the hypothesis and size of the mammal according to the literature was not adequate to get accurate physiologic responses.

According to our experimental study results, we concluded that cooled medical oxygen technique seems to be as effective as the currently widely accepted TH techniques. As an easy-to-use, reversible, and inexpensive method, cooled medical oxygen inhalation may be a beneficial hypothermia technique, specifically in the early phases of TH. It can also be combined with current hypothermia techniques for better results; further studies seem promising.

## Figures and Tables

**Table 1:**
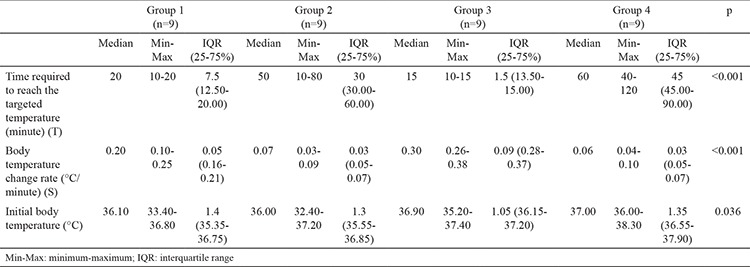
Cooling rates for all of the study groups

**Table 2:**
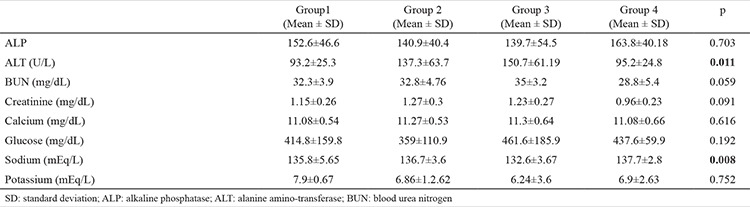
Biochemical analyses of study groups and control group
